# Establishment and optimization of a tobacco rattle virus -based virus-induced gene Silencing in *Atriplex canescens*

**DOI:** 10.1186/s13007-025-01427-z

**Published:** 2025-08-07

**Authors:** Shan Feng, Jin-Da Chen, Ai-Ke Bao

**Affiliations:** State Key Laboratory of Herbage Improvement and Grassland Agro-Ecosystems, Key Laboratory of Grassland Livestock Industry Innovation, Ministry of Agriculture and Rural Affairs, College of Pastoral Agriculture Science and Technology, Lanzhou University, Lanzhou, 730000 China

**Keywords:** Virus-induced gene silencing, Tobacco rattle virus, *Atriplex canescens*

## Abstract

*Atriplex canescens*, a halophytic model plant exhibiting remarkable tolerance to abiotic stress, possesses valuable genetic resources for crop improvement. However, the absence of a reliable and stable transformation systems has impeded the functional characterization of candidate genes in this species. In this study, we established an efficient virus-induced gene silencing (VIGS) system in *A. canescens* by constructing a tobacco rattle virus (TRV)-based vector targeting the phytoene desaturase gene (*AcPDS*), whose silencing induces an easily recognizable photobleaching phenotype. Through comparative analyses of inoculation materials and methods for VIGS implementation in *A. canescens*, we found that inoculating germinated seeds with *Agrobacterium* suspension (OD_600_ = 0.8) carrying TRV2:*AcPDS* vectors through vacuum-assisted agroinfiltration (0.5 kPa, 10 min) further achieving an average silencing efficiency of approximately 16.4%. Systemic photobleaching phenotypes appeared in newly emerged leaves at about 15 days post-inoculation, accompanied by a 40–80% reduction in *AcPDS* transcript abundance, as confirmed by qRT-PCR. Furthermore, we successfully validated this VIGS system by silencing two aquaporin genes (*AcTIP2;1* and *AcPIP2;5*), achieving 60.3–69.5% knockdown efficiency, which confirmed the broad applicability of this system in *A. canescens*. In summary, our method provides a reliable reverse-genetics platform that facilitates the study of stress-resistance mechanisms in *A. canescens*, effectively overcoming previous technical limitations in genetic research on this species.

**Supplementary Information:**

The online version contains supplementary material available at 10.1186/s13007-025-01427-z.

## Introduction

*Atriplex canescens* is a perennial halophytic shrub widely recognized for its exceptional adaptability to diverse harsh environments, and is extensively grown in saline-alkali and desert regions [1–3]. Our previous transcriptomic analysis revealed that numerous genes were significantly upregulated in *A. canescens* under salt treatment, suggesting the presence of substantial genetic resources related to stress resistance in this species [4, 5]. However, functional characterization of these genes has been hindered by the lack of an efficient genetic transformation system. While we previously attempted to develop a stable transformation protocol for *A. canescens* by infecting in vitro cultured apical meristems, challenges such as low efficiency and chimeric outcomes persisted. Conventional reverse genetics approaches, such as RNA interference (RNAi), T-DNA insertional mutagenesis, and CRISPR/Cas9-mediated gene editing, have revolutionized functional genomics in model plants [6]. Unfortunately, these methods typically rely heavily on stable genetic transformation to generate loss-of-function mutants [7–10], making them impractical for *A. canescens*.

Virus-induced gene silencing (VIGS) utilizes viruses to specifically downregulate endogenous gene expression through post-transcriptional gene silencing (PTGS) triggered upon viral infection in host cells [11–13]. This approach is particularly advantageous for non-model plant species, as it provides a rapid and efficient alternative to traditional genetic transformation procedures, which are often laborious, time-consuming, and challenging, especially in plants with inherently low transformation efficiency [13–18]. The versatility of VIGS is demonstrated by its successful implementation across diverse plant systems using various viral vectors, including tobacco rattle virus (TRV) for disease resistance studies in cotton [19], barley stripe mosaic virus (BSMV) and cucumber mosaic virus (CMV) for functional genomics in cereal crops [20], as well as applications in vegetable crops like tomato and pepper for developmental research [21]. Among these, the TRV vector system (pTRV2 and pTRV1) has become an effective tool due to its extensive host range and ease of use [22], as evidenced by successful establishments in wheat [23], *Lycium barbarum* [24], *Miscanthus* [10] and *Camellia drupifera* [18]. However, the applicability and effectiveness of VIGS vectors for functional genetic studies in *A. canescens* remains unclear.

Phytoene desaturase (PDS) is a key rate-limiting enzyme in carotenoid biosynthesis [25, 26]. The disruption of *PDS* gene expression in plants typically results in albino or photobleaching phenotype, making *PDS* a widely utilized endogenous reporter gene for assessing the effectiveness of gene silencing systems [27]. In this study, we established a TRV-based VIGS system in *A. canescens* using the endogenous marker genes *AcPDS*. Upon silencing *AcPDS*, we evaluated the feasibility and efficiency of the established VIGS-TRV system through phenotypic observations and quantitative analyses. Furthermore, we optimized this system by modifying infection materials and inoculation methods. As a result, we successfully developed an efficient and user-friendly VIGS-TRV system, providing a valuable methodological platform for functional gene investigation in *A. canescens*.

## Materials and methods

### Plant materials

Seeds of *A. canescens* were collected from plants cultivated in Minqin County (38°37′, 103°5′; elevation 1400 m), Gansu province, China. To facilitate seed germination through weakening the hard seed coat, the collected seeds were immersed in 50% (v/v) H_2_SO_4_ for 8 h. Following acid treatment, seeds were thoroughly rinsed with distilled water until no acid odor was detectable, then placed on moistened vermiculite in petri dishes and incubated at 25 °C in darkness to initiate germinate. Germinated seeds exhibiting radicle lengths of approximately 1–3 cm were selected for subsequent *Agrobacterium*-mediated inoculation experiments.

### Identification and cloning of *AcPDS*, *AcTIP2;1* and *AcPIP2;5* gene from *A. canescens*

To identify and clone the full-length open reading frame (ORF) of the *AcPDS*,* AcTIP2;1* and *AcPIP2;5* gene from *A. canescens*, we performed sequence searches against the National Center for Biotechnology Information (NCBI) database using the Basic Local Alignment Search Tool (https://blast.ncbi.nlm.nih.gov/Blast.cgi), guided by our previously obtained transcriptome sequencing data (Wang and Bao, unpublished data). The complete *AcPDS*,* AcTIP2;1* and *AcPIP2;5* ORF sequence was amplified using nested PCR according to the reaction mixture and cycling conditions described by Feng et al. [5]. The primers for the nested PCR amplification of the *AcPDS*,* AcTIP2;1* and *AcPIP2;5* cDNA are detailed in Supplementary Table S1.

### Construction of TRV-VIGS vectors

To select gene fragments with high silencing efficiency potential, we utilized the SGN-VIGS online tool (https://vigs.solgenomics.net/) for predicting optimal nucleotide target regions [28]. Three conserved *AcPDS* gene fragments ranging from 300 to 400 bp were selected, corresponding respectively to the 5′ end, central region, and 3′ end of the *AcPDS* ORF. Then Nucleotide-BLAST was used to verify sequence specificity, and highly specific fragments were selected as target fragments. These selected gene fragments were amplified from *A. canescens* cDNA using primers containing *EcoRI* and *BamHI* restriction enzyme recognition sites (Supplementary Table S1). The amplified gene fragments were subsequently cloned into the pTRV2 vector, generating three recombinant VIGS vectors designated as TRV2:*PDS*_*311*_, TRV2:*PDS*_*751*_, TRV2:*PDS*_*1221*_.

For functional validation of the VIGS system, gene-specific fragments of *AcTIP2;1* (325 bp) and *AcPIP2;5* (428 bp) were amplified using primers listed in Supplementary Table S1. These fragments were cloned into containing *EcoRI* and *BamHI* restriction enzyme recognition sites of the pTRV2-GFP vector, which retains the original TRV2 backbone but incorporates an enhanced GFP reporter downstream of the CaMV 35 S promoter for visual screening [29].

### Preparation of *Agrobacterium* infection suspension

To establish an efficient TRV-based VIGS system in *A. canescens*, recombinant vector plasmids (TRV2:0, TRV2:*PDS*_*311*_, TRV2:*PDS*_*751*_, TRV2:*PDS*_*1221*_) and TRV1 were introduced into *A. tumefaciens* strain GV3101 via freeze-thaw transformation. Transformed bacteria cells were plated onto YEP agar containing 50 mg/L kanamycin and 50 mg/L rifampicin, followed by incubation at 28 °C for 48 h. Individual colonies were inoculated into 100 mL YEP liquid medium supplemented with the same antibiotics and cultured at 28 °C with 200 rpm shaking until reaching the mid-logarithmic growth phase (OD_600_ = 0.6–0.8, approximately 5–6 h). The value of OD_600_ value was determined using a spectrophotometer (UV-6100PCS; Mapada Instrument, China). Subsequently, bacterial cells were collected by centrifugation at 6000 rpm for 8 min and resuspended in infiltration buffer (10 mM 2-[N-Morpholino] ethane sulfonic acid (MES), 200 µM acetosyringone (AS), 10 mM MgCl_2_, 0.03% Silwet-77) to an OD_600_ between 0.8 and 1.0, as described by Zhang et al. [30]. Equal volumes of TRV1- and TRV2-derived *Agrobacterium* suspensions were combined and incubated at room temperature in darkness for 3 h prior to inoculation, thus inducing expression of virulence genes [31].

### TRV inoculation into *A. canescens*

To optimize TRV-mediated gene silencing efficiency in *A. canescens*, the germinated seeds were divided into two groups for infection experiments: (1) intact seeds with seed coats, and (2) decorticated seeds exposing folded cotyledons. Each group was subjected to TRV inoculation via either soaking or vacuum-assisted agroinfiltration.

For the soaking method, experimental materials were immersed in an *Agrobacterium* infiltration suspension containing either TRV1 + TRV2:*AcPDS* (experimental) or TRV1 + TRV2:0 (empty vector) for 40 min, with gentle shaking maintained at 50 rpm. After inoculation, residual bacterial suspension was removed by rinsing three times with sterile distilled water. The inoculated materials were then transplanted into individual pots (10 cm diameter) filled with vermiculite and grown in a greenhouse under controlled conditions of 22 °C, 16 h day/8 h night cycle, and a lighting intensity of 150 µM m^− 2^ s^− 1^. Plants were irrigated weekly with 1/2-strength Hoagland nutrient solution to maintain optimal growth.

For vacuum-assisted agroinfiltration, the prepared materials were individually submerged in *Agrobacterium* suspension containing either TRV1 + TRV2:*AcPDS* or TRV1 + TRV2:0. Materials were subjected to two cycles of vacuum infiltration (0.5 kPa for 5 min per cycle, 10 min total) in a vacuum chamber (AP-9925 N, China). Following vacuum infiltration, the materials were co-cultivated in the same bacterial suspension for an additional 40 min with gentle agitation (50 rpm). Finally, the inoculated materials were rinsed three times with sterile distilled water to remove surface- bound bacteria cells and transferred into individual pots (10 cm diameter) filled with vermiculite under the same greenhouse cultivation conditions as described for the soaking method.

For the expression validation of *AcTIP2;1* and *AcPIP2;5*, prepared plant materials were separately immersed in *Agrobacterium* suspensions containing either TRV1 + TRV2:0 (empty vector control) or TRV1 + TRV2-GFP:*AcTIP2;1*, TRV1 + TRV2-GFP:*AcPIP2;5* constructs. The inoculation was performed using the previously described vacuum infiltration protocol (0.5 kPa for 5 min per cycle, 10 min total). Following 15 days of post-infiltration cultivation under same growth conditions as described for the soaking method, systemic infection was monitored using a 100 W hand-held long-wave ultraviolet lamp (UV products, USA; Black Ray model B 100AP/R) [29]. Only plant tissues exhibiting GFP fluorescence coverage exceeding 80% were collected for subsequent qRT-PCR analysis to ensure reliable assessment of gene silencing efficiency.

### Total RNA extraction and qRT-PCR validation

To evaluate the silencing efficiency and confirm *AcPDS*,* AcTIP2;1* and *AcPIP2;5* transcript knockdown, total RNA was extracted from the leaves of wild type (WT, non-infected control), TRV2:0, TRV2:*AcPDS*-infected plants exhibiting photobleaching phenotypes, TRV2:*AcTIP2;1*-infected plants and TRV2:*AcPIP2;5*-infected plants, using TaKaRa Mini BEST Plant RNA Extraction Kit (TaKaRa, Japan). First-strand cDNA synthesis was performed using the PrimeScript™ II first strand cDNA Synthesis Kit (TaKaRa, Japan), according to the manufacturer’s protocol. Primers used for qRT-PCR analysis are listed in Supplementary Table S2. The *AcActin* gene from *A. canescens* served as an internal reference. The qRT-PCR was conducted in 20 µL reactions containing SYBR Green Real-Time PCR Master Mix (TaKaRa, Japan) on a StepOnePlus Real-Time PCR System (Thermo Scientific, USA), with three biological replicates per treatment. Relative expression levels of *AcPDS* were calculated using the 2^−ΔΔCt^ method [32].

### Determination of chlorophyll content

To quantitatively assess the photobleaching phenotype resulting from gene silencing, the chlorophyll (chlorophyll *a* and chlorophyll *b*) content was measured in leaves of control plants (WT and TRV2:0) and TRV2:*AcPDS*-infected plants following the method described by Chen et al. [33], with slight modifications.

### Statistical analysis

The data analyses were performed using SPSS software (version 22.0; IBM, USA). Significant differences among treatments were determined using one-way analysis of variance (ANOVA) followed by Duncan’s multiple range tests, with statistical significance defined at *P* < 0.05. All data are expressed as mean ± standard error of the mean (SEM) from three independent experimental data.

## Results

### Identification of *AcPDS* and construction of pTRV:*AcPDS* vectors

**Fig. 1:**
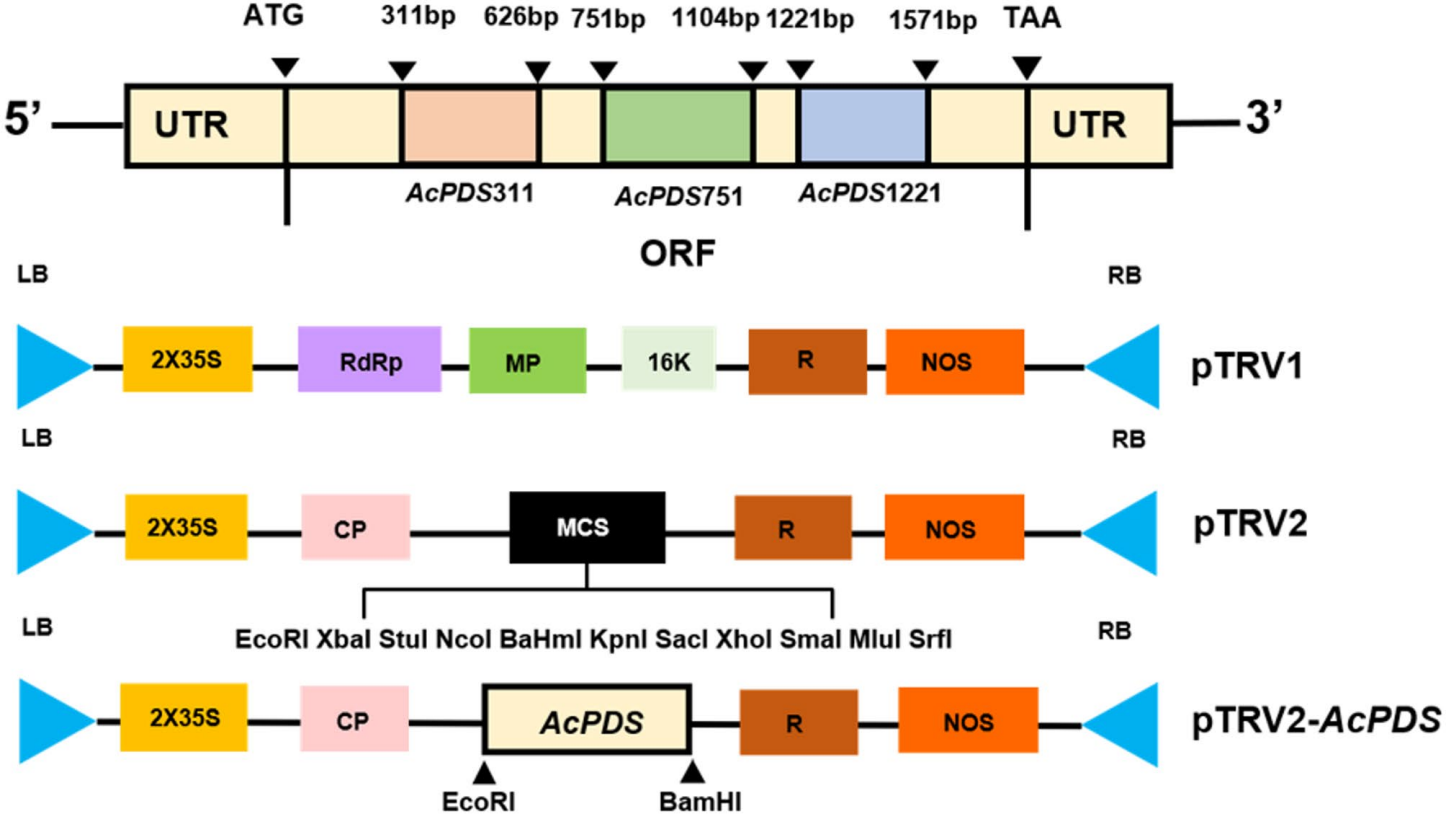
Schematic diagram illustrating the construction of the TRV2:*AcPDS* recombinant vector. Numbers indicate nucleotide positions corresponding to the open reading frame (ORF). MP: movement protein; 16 K: 16 kDa protein-encoding fragment; CP: coat protein; MCS: multiple cloning sites; R:self-cleaving ribozyme; NOS: nopaline synthase terminator; LB and RB: left and right borders; 2 × 35 S: double CaMV 35 S promoter

The full-length sequence of *AcPDS* was obtained through nested-PCR based on our previously generated transc riptome data of *A. canescens* (Wang and Bao, unpublished data) (Supplementary Fig. S1A, B). Multiple sequence alignments demonstrated that the deduced amino acid sequence of AcPDS shared high similarity with orthologous proteins from *Chenopodium quinoa* (XP_021761845.1), *Spinacia oleracea* (XP_021847448.1), and *Beta vulgaris* (XP_010685188.1) (Supplementary Fig. S1C). Specifically, sequence identity between AcPDS and CqPDS reached 96.3%, and phylogenetic analysis revealed that AcPDS clustered closely with CqPDS in a distinct clade, indicating their close evolutionary relationship (Supplementary Fig. S1D). To achieve targeted silencing of *AcPDS* expression in *A. canescens*, we amplified three highly conserved fragments, including *AcPDS*_*311*_, *AcPDS*_*751*_ and *AcPDS*_*1221*_ from different regions of the *AcPDS* ORF and subsequently cloned into the TRV vector, generating recombinant silencing vectors TRV2:*AcPDS*_*311*_, TRV2:*AcPDS*_*751*_ and TRV2:*AcPDS*_*1221*_ (Fig. 1).

### Silencing of *AcPDS* in germinated seeds of *A. canescens* via *Agrobacterium* soaking

**Fig. 2:**
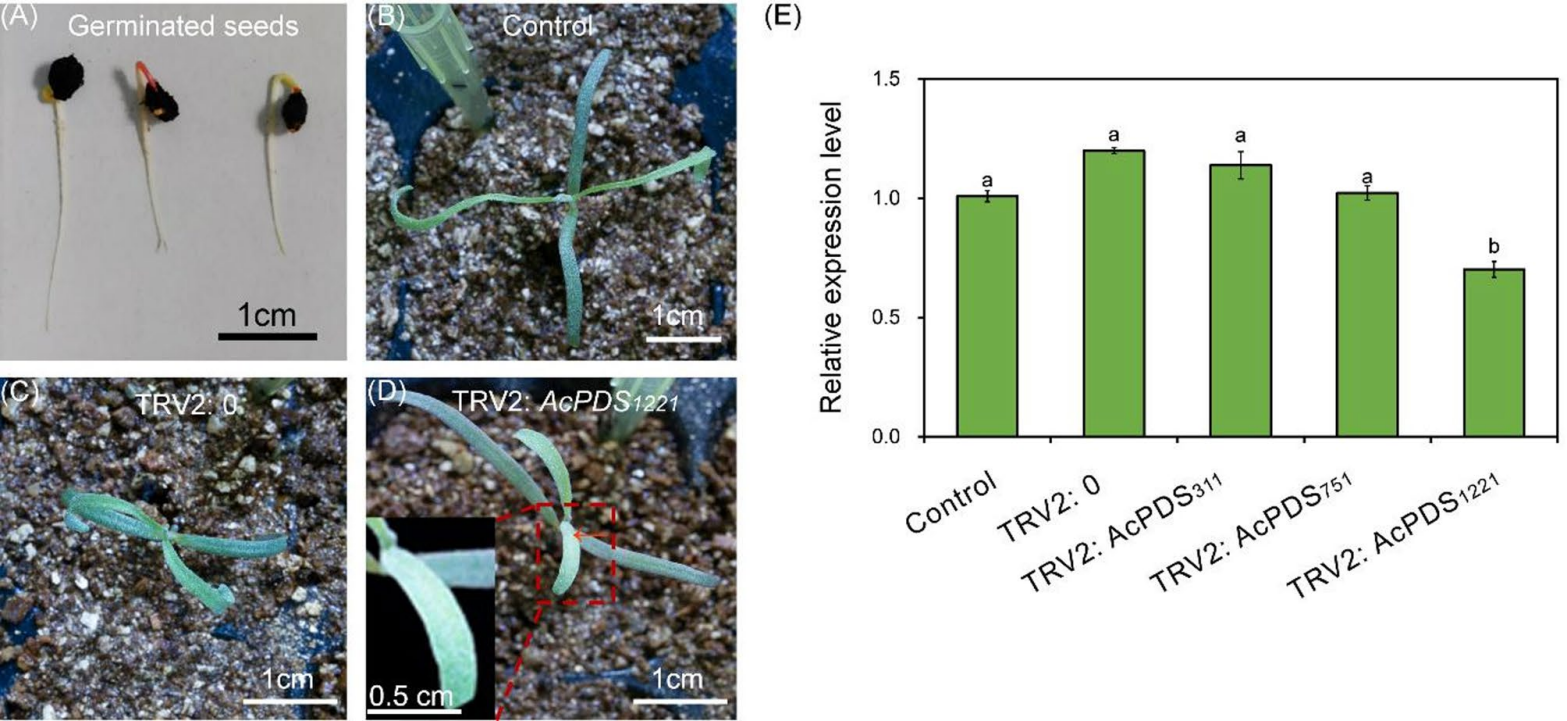
Phenotypic and molecular characterization of *A. canescens* seedlings at 15 days post-inoculation via *Agrobacterium* soaking method. (**A**) Germinated seeds utilized as inoculation material. (**B**) Non-inoculated control seedlings. (**C**) Seedlings inoculated with *Agrobacterium* carrying the empty TRV2 vector (TRV2:0). (**D**) Seedlings inoculated with *Agrobacterium* harboring TRV2:*AcPDS*_*1221*_, exhibiting a photobleaching phenotype. (**E**) Relative expression levels of *AcPDS* gene in control (TRV2:0) and TRV2:*AcPDS*-infected seedlings. TRV2:*AcPDS*_*311*_, TRV2:*AcPDS*_*751*_, and TRV2:*AcPDS*_*1221*_ represent recombinant VIGS vectors containing different conserved *AcPDS* fragments. Scale bars in (**A**)-(**D**) are equal to 1 cm. Different letters in (**E**) indicate significant differences at *P* < 0.05 (Duncan’s test). Values are means ± SE (*n* = 3)

To evaluate the efficacy of recombinant TRV vectors in mediating *AcPDS* silencing, germinating seeds of *A. canescens* were infected using *Agrobacterium* soaking method (Fig. 2A). At 15 days post-inoculation, only newly emerged buds and leaves of seedlings inoculated with TRV2:*AcPDS*_*1221*_ exhibited mild photobleaching phenotypes, while seedlings treated with the empty vector (TRV2:0) or untreated controls displayed normal green coloration under identical growth conditions (Fig. 2B-C). The qRT-PCR analysis indicated that *AcPDS* transcript abundance decreased by approximately 30% in photobleached leaves from TRV2:*AcPDS*_*1221*_-infected plants compared to both control and TRV2:0 seedlings (Fig. 2D). These results confirmed that the recombinant TRV vector successfully induced gene silencing in *A. canescens*.

### Silencing of *AcPDS* in germinated seeds of *A. canescens* via vacuum-assisted agroinfiltration

**Fig. 3:**
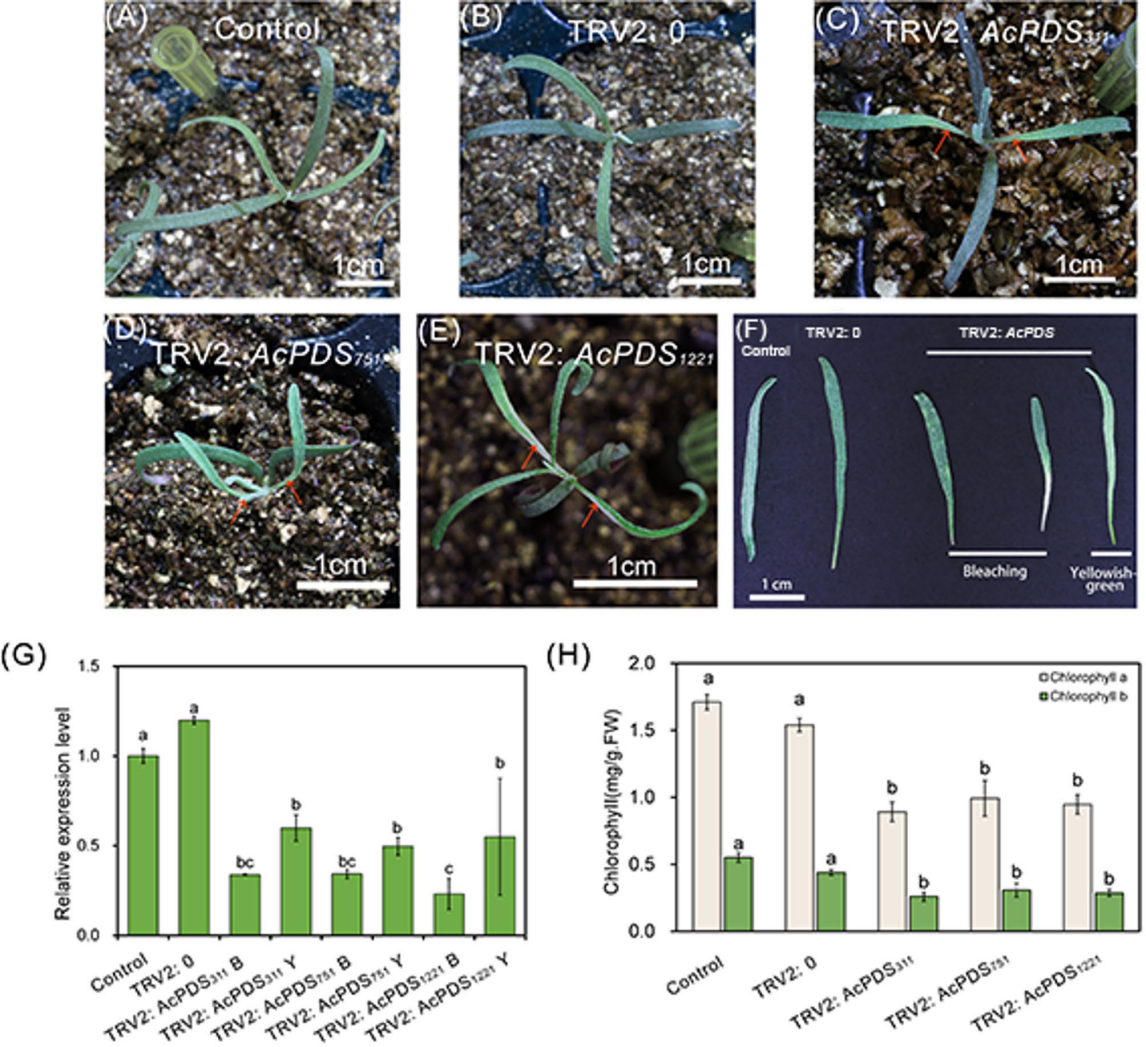
Phenotypic and molecular characterization of *A. canescens* seedlings at 15 days post-inoculation via vacuum-assisted vacuum-assisted agroinfiltration. (**A**) Non-inoculated control seedlings. (**B**) Seedlings inoculated with *Agrobacterium* carrying the empty TRV2 vector (TRV2:0). (**C**)-(**E**) Seedlings inoculated with *Agrobacterium* harboring TRV2:*AcPDS*_*311*_, TRV2:*AcPDS*_*751*_, and TRV2:*AcPDS*_*1221*_, respectively. (**F**) Comparison of leaf phenotypes resulting from *AcPDS* silencing. (**G**) Relative *AcPDS* expression levels in control and TRV2:*AcPDS*-infected seedlings. “B” and “Y” represents fully bleached and yellowish-green, respectively. (**H**) Chlorophyll *a* and chlorophyll *b* contents in leaves of control and *AcPDS*-silenced *A. canescens* plants. TRV2:*AcPDS*_*311*_, TRV2:*AcPDS*_*751*_, and TRV2:*AcPDS*_*1221*_ represent recombinant VIGS vectors containing different conserved *AcPDS* fragments. Scale bars in (**A**)-(**F**) are equal to 1 cm. Different letters in (**G**) and (**H**) indicate significant differences at *P* < 0.05 (Duncan’s test). Values are means ± SE (*n* = 3)

We subsequently investigate the effectiveness of *AcPDS* silencing using vacuum-assisted agroinfiltration. As shown in Fig. 3A-F, typical albino phenotype consistently appeared at the basal regions of newly developed leaves on TRV2:*AcPDS*-infected seedlings at 15 days post-inoculation, whereas no photobleaching was observed in control and TRV2:0 seedlings. The qRT-qPCR data demonstrated significant suppression of *AcPDS* expression in TRV2:*AcPDS*-infected seedlings, with transcript reductions ranging from 65% to 80% in fully bleached leaves to approximately 40%-58% in yellowish-green leaves compared to both control groups (Fig. 3G). Comparative analysis among three independent TRV2:*AcPDS* constructs (TRV2:*AcPDS*_*311*_, TRV2:*AcPDS*_*751*_, and TRV2:*AcPDS*_*1221*_) indicated silencing efficiencies of 21.1%, 12.4%, and 15.6%, respectively, as evidenced by the proportion of seedlings exhibiting clear photobleaching phenotypes (Fig. S2). Consistent with transcript suppression, chlorophyll content analysis revealed corresponding reductions in photosynthetic pigments, with chlorophyll *a* decreased by 35–47% and chlorophyll *b* by 30–52% in TRV2:*AcPDS*-infected seedlings relative to control and TRV2:0 plants (Fig. 3F).

### Silencing of *AcPDS* in folded cotyledons of *A. canescens* via vacuum-assisted agroinfiltration

**Fig. 4:**
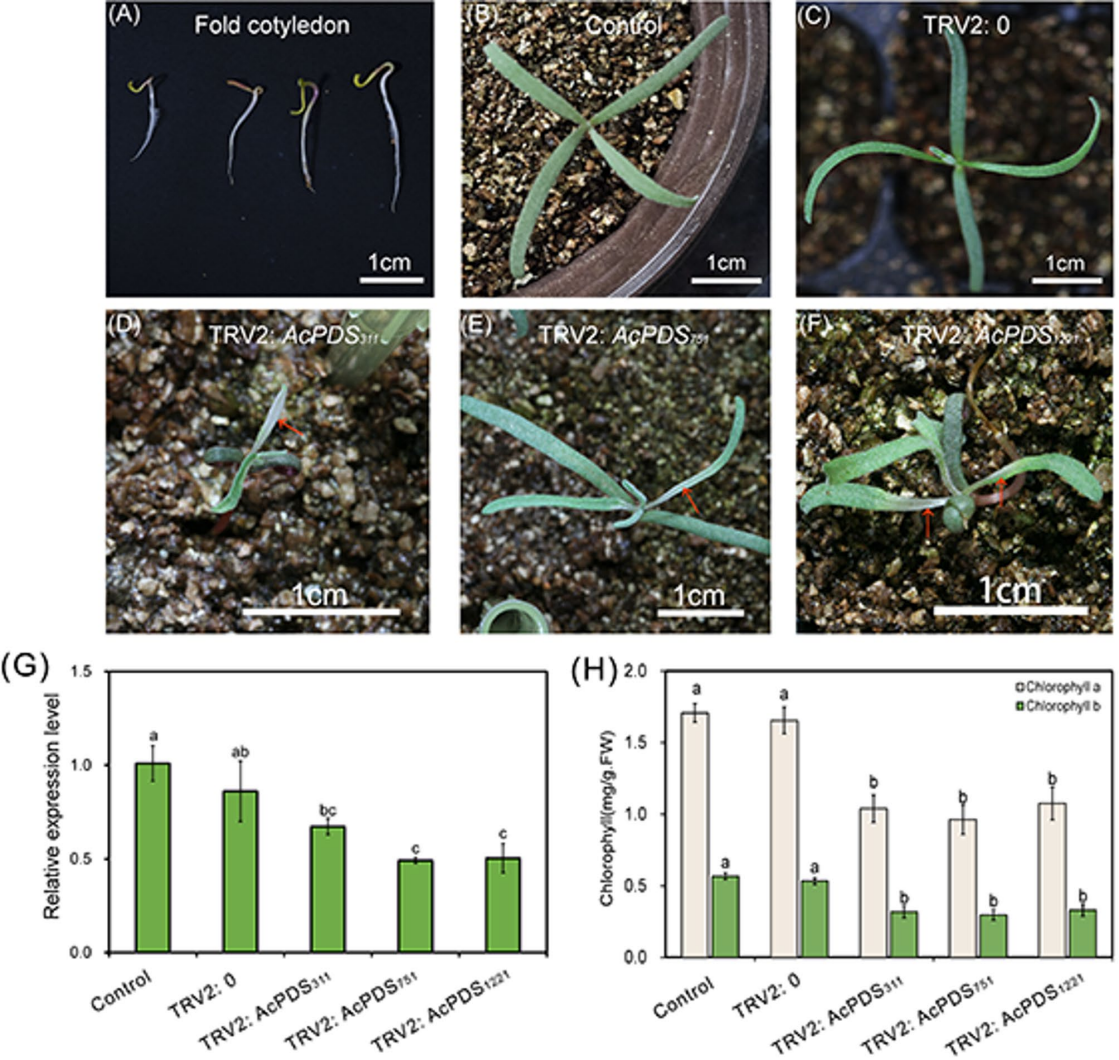
Phenotypic and molecular characterization of *A. canescens* seedlings at 15 days post-inoculation via vacuum-assisted agroinfiltration of folded cotyledons. (**A**) Germinated seeds with seed coats removed to expose folded cotyledons (inoculation material). (**B**) Non-inoculated control seedlings. (**C**) Seedlings inoculated with *Agrobacterium* carrying the empty TRV2 vector (TRV2:0). (**D**)-(**F**) Seedlings inoculated with *Agrobacterium* harboring TRV2:*AcPDS*_*311*_, TRV2:*AcPDS*_*751*_, and TRV2:*AcPDS*_*1221*_, respectively. (**G**) Relative *AcPDS* expression levels in control and TRV2:*AcPDS*-infected seedlings. (**H**) Chlorophyll *a* and chlorophyll *b* contents in leaves of control and *AcPDS*-silenced *A. canescens* plants. TRV2:*AcPDS*_*311*_, TRV2:*AcPDS*_*751*_, and TRV2:*AcPDS*_*1221*_ represent recombinant VIGS vectors containing different conserved *AcPDS* fragments. Scale bars in (**A**)-(**F**) are equal to 1 cm. Different letters in (**G**) and (**H**) indicate significant differences at *P* < 0.05 (Duncan’s test). Values are means ± SE (*n* = 3)

Preliminary trials indicated that cotyledon-stage seedlings of *A. canescens* possess elongated, fragile hypocotyls highly susceptible to mechanical injury during vacuum-assisted agroinfiltration, resulting in reduced seedling survival rate. To overcome this limitation, *A. canescens* seeds germinated for 4 days on vermiculite were manually decorticated to expose folded cotyledons, which were subsequently subjected to vacuum-assisted agroinfiltration with *Agrobacterium* carrying the TRV2:*AcPDS* vector (Fig. 4A). Phenotypic analysis at 15 days after infiltration revealed characteristic photobleaching in leaves of TRV2:*AcPDS*-infected seedlings, with a silencing efficiency estimated at approximately 14.7% (Fig. 4B-F, Fig. S3A). However, this infiltration procedure negatively affected seedling viability, causing approximately 30% reduction in survival rates for both TRV2:*AcPDS* and TRV2:0 groups compared with untreated control seedlings (Supplementary Fig. S3B). The qRT-PCR analysis demonstrated that *AcPDS* transcript levels in TRV2:*AcPDS* plants were reduced by 21–51% compared to control and TRV2:0 plants (Fig. 4G). Consistent with partial silencing effects, spectrophotometric measurements confirmed significant reductions in photosynthetic pigment levels, with chlorophyll *a* decreasing by 35%-43% and chlorophyll *b* decreasing by 37%-44% in TRV2:*AcPDS*-infected plants compared to both control and TRV2:0 (Fig. 4H).

### Validation of VIGS efficiency using Aquaporin genes *AcTIP2;1* and *AcPIP2;5*

**Fig. 5:**
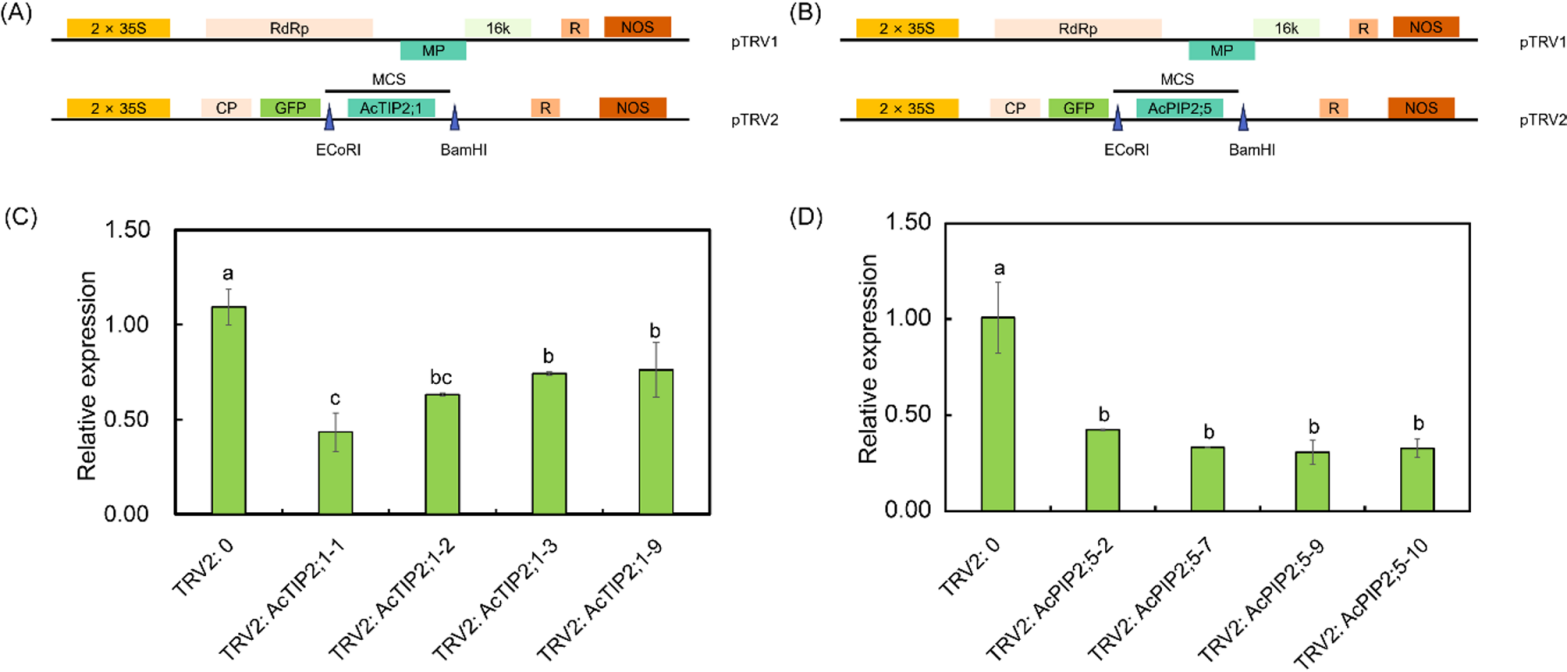
Molecular characterization and silencing efficiency of *AcTIP2;1* and *AcPIP2;5* in *A. canescens*. Schematic representation of the TRV2:*AcTIP2;1* vector (**A**) and TRV2:*AcPIP2;5* vector construct (**B**). Relative expression levels of *AcTIP2;1* in TRV2:0 (empty vector control) and TRV2:*AcTIP2;1* (**C**) and *AcPIP2;5* in TRV2:0 and TRV2:*AcPIP2;5* at 15 days post-inoculation (dpi) (**D**). Different letters in (**C**) and (**D**) indicate significant differences at *P* < 0.05 (Duncan’s test). Values are means ± SE (*n* = 3)

To further validate the efficacy of the VIGS system in *A. canescens*, we successfully cloned two aquaporin genes, including *AcTIP2;1* and *AcPIP2;5*, and constructed their respective TRV-based silencing vectors (Fig. 5A and B). The recombinant vectors were introduced into germinated seeds through vacuum-assisted agroinfiltration (OD600 = 0.8-1.0, 10 min vacuum infiltration followed by 40 min co-cultivation). At 15 days after infiltration, four transgenic lines exhibiting GFP fluorescence coverage exceeding 80% were selected from ten candidate lines for qRT-PCR analysis to assess gene silencing efficiency. The results showed that compared to the TRV2 empty vector (TRV2:0), the transcript levels of *AcTIP2;1* and *AcPIP2;5* were significantly reduced by 30.2–60.3 and 58.0-69.5%, respectively (Fig. 5C and D). These results demonstrate that our optimized VIGS protocol achieves robust and functional gene knockdown in *A. canescens*.

## Discussion

TRV-mediated VIGS system has emerged as a powerful high-throughput tool for functional gene analysis in plants that circumvents the need for stable genetic transformation [34–36]. While achieving 70% higher silencing efficiency in model species like *Nicotiana benthamiana* and tomato, its application in stress-adapted species with specialized morphologies remains challenging [37]. In this study, we successfully adapted the TRV-based VIGS system to the halophytic species *A. canescens* (Fig. 2). More importantly, we selected *AcPDS* as our visual reporter gene due to its characteristic photobleaching phenotype upon silencing [37]. Similar strategies utilizing *PDS* as a reporter gene have been successfully employed in other plant species, including *Areca catechu* and *Luffa acutangula*, to optimize VIGS protocols [38, 39]. Although the observed silencing efficiency was relatively lower than some previously reported in other species, this discrepancy likely reflects several biological and technical constraints [40]. On the one hand, TRV’s host range limitations may affect viral spread in *A. canescens*, as many halophytes exhibit enhanced antiviral defense mechanisms [41]. On the other hand, the design of the VIGS vector, including the choice and orientation of the target gene fragment, may not be optimal for efficient silencing in *A. canescens*. For instance, the length and sequence characteristics of the *AcPDS* fragment used in the vector, as well as the direction of insertion, could affect the silencing outcome [42]. Therefore, optimizing the VIGS vector design and exploring alternative VIGS systems tailored for halophytes may be necessary to improve the silencing efficiency in *A. canescens* in the future.

Efficient delivery of viral vectors into plant tissues is a critical determinant of VIGS success [10, 43]. In dicotyledonous plants, common VIGS inoculation methods include needleless syringe infiltration and vacuum-assisted agroinfiltration of leaves [44–47]. However, needleless syringe infiltration proved challenging in *A. canescens* due to its narrow, elongated leaves, even when the smallest available syringes were employed. Furthermore, our attempts to infect cotyledon-stage seedlings and decapitated seedlings of *A. canescens* via vacuum-assisted agroinfiltration were unsuccessful, resulting in low silencing efficiencies and significantly reduced seedling survival rates.

Apart from leaf infiltration methods, previous studies have demonstrated that *Agrobacterium*-mediated co-cultivation of germinating seeds represents an efficient VIGS inoculation alternative in certain plant species [10, 23]. For instance, Zhang et al. [48] successfully induced gene silencing by soaking germinating cotton seeds without seed coats in an *Agrobacterium* suspension containing TRV-VIGS vectors. Adapting this seed-based inoculation approach, we successfully achieved gene silencing in germinated seeds of *A. canescens*, as evidenced by qRT-PCR analysis showing a significant 30% reduction in *AcPDS* transcript abundance, thus validating the suitability of this method for this species (Fig. 2). Moreover, vacuum-assisted agroinfiltration has been reported to markedly enhance VIGS efficiency in numerous plant species. For example, Zhang et al. [23] achieved whole-plant photobleaching in germinating wheat and scarified maize seeds using vacuum infiltration, while Liu et al. [49] reported optimal silencing efficiencies in eggplant seedlings vacuum-infiltrated at -25 kPa for 2 min. In our study, vacuum-assisted agroinfiltration resulted in a maximum silencing efficiency of 21.1%, with qRT-PCR analysis indicating a pronounced reduction (40–80%) in *AcPDS* transcript levels in TRV2:*AcPDS*-infected seedlings exhibiting typical photobleaching phenotypes (Fig. 3). Notably, this system achieved higher silencing efficiencies (30.2–60.3 for *AcTIP2;1* and 58.0-69.5% for *AcPIP2;5*) for aquaporin genes compared to the *AcPDS* reporter (Fig. 5). These results strongly suggest that vacuum-assisted agroinfiltration is more effective for achieving VIGS in *A. canescens* compared to conventional soaking methods, likely due to improved penetration of *Agrobacteria* suspensions into epidermal cells under vacuum conditions.

Previous studies have indicated that removal of seed coat can significantly enhance VIGS efficiency by facilitating *Agrobacterium* penetration into seed tissues [23, 48]. However, in contrast to these earlier reports, our results showed that decorticated seeds (folded cotyledons) did not significantly improve VIGS efficiency compared to intact seeds in *A. canescens* (Fig. 4). This discrepancy may reflect species-specific differences, possibly because seeds at identical developmental stages (with or without coats) exhibited comparable susceptibility to *Agrobacterium* infection. Moreover, the vacuum infiltration parameters currently employed may require further refinement, as evidenced by the parallel unsuccessful attempts to infect decapitated seedlings under similar conditions. Additionally, we found that the position of the target fragment within the *AcPDS* gene did not significantly impact silencing efficiency in our system. Overall, our results clearly demonstrate that germinated seeds represent the most suitable material for TRV-mediated VIGS experiments in *A. canescens*, surpassing alternative tissue types and inoculation methods tested in this study.

## Conclusion

**Fig. 6:**
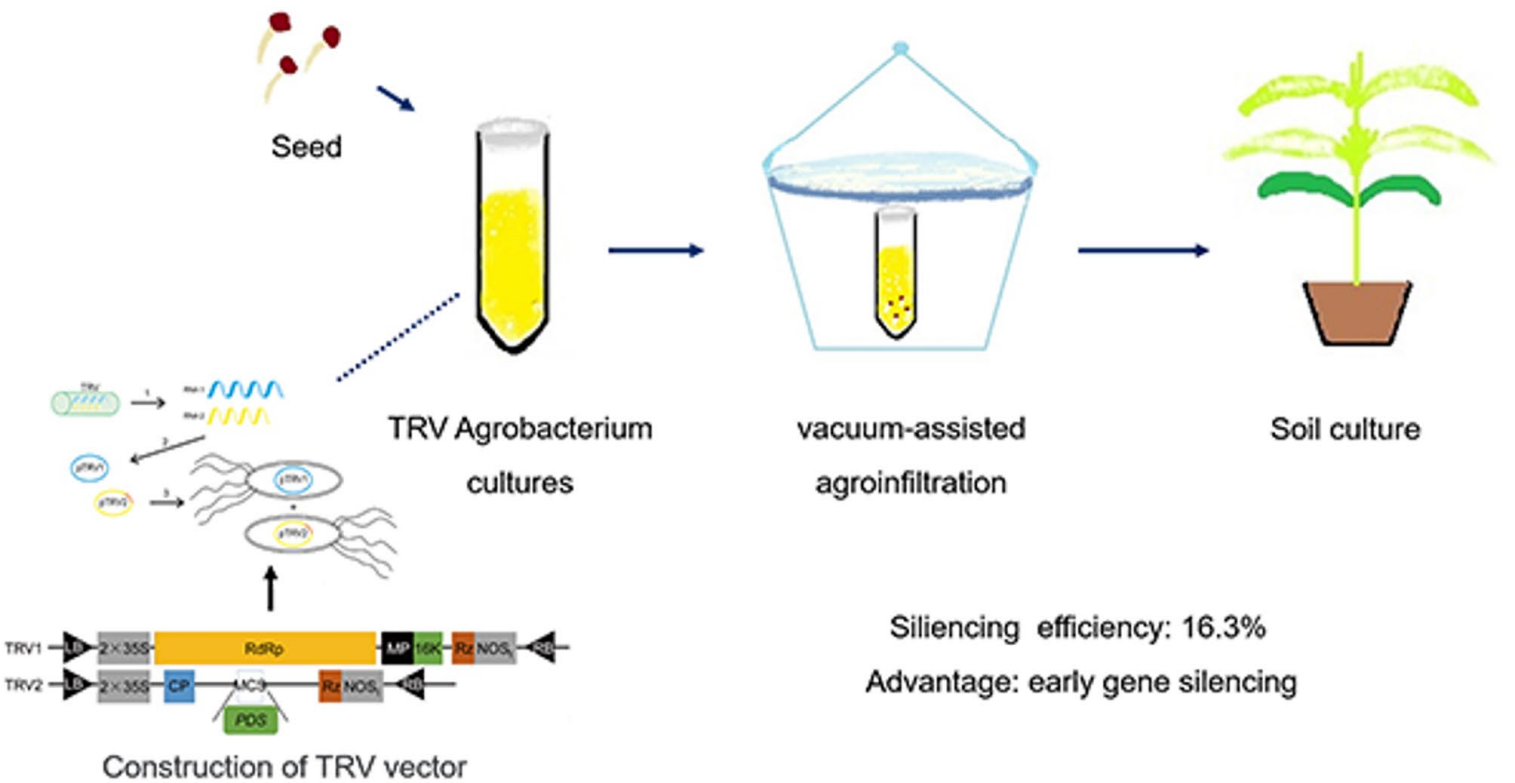
Workflow outlining the establishment of a TRV-VIGS system in *A. canescens*. This procedure includes: (1) construction of TRV1 and TRV2 recombinant vectors; (2) preparation of recombinant *Agrobacterium* suspension (OD_600_ = 0.8); and (3) vacuum-assisted agroinfiltration of 2-day-old germinated seeds, ultimately achieving a gene silencing efficiency of approximately 16.3% silencing efficiency

In this study, we successfully isolated and cloned the full-length cDNA of *AcPDS* from *A. canescens*. A 300–350 bp conserved fragment of the *AcPDS* gene was selected to construct the TRV2:*AcPDS* vector, resulting in effective suppression (40–80%) of target gene transcript levels following optimized vacuum-assisted agroinfiltration conditions (OD_600_ = 0.8-1.0, 10 min vacuum infiltration followed by 40 min co-cultivation). This optimized VIGS protocol achieved an average silencing efficiency of approximately 16.4% in germinated seeds, providing a valuable foundation for future functional genomics studies in *A. canescens* (Fig. 6).

## Supplementary Information

Below is the link to the electronic supplementary material.

**Supplementary Material 1: Supplementary Fig. S1**: Isolation and characterization of the *AcPDS* gene from *A. canescens*. (A) Amplification of the full-length *AcPDS* cDNA sequence by nested PCR. (B) Amplification of three conserved *AcPDS* target fragments. (C) Multiple alignment of AcPDS protein with its homologs from other species. (D) Phylogenetic analysis of AcPDS and related homologs. **Supplementary Fig. S2**: Comparison of silencing efficiencies and seedling survival rates at 15 days post-inoculation of germinated *A. canescens* seeds via vacuum infiltration. (A) Silencing efficiency of TRV2:*AcPDS*-infected plants (TRV2:*AcPDS*_*311*_, *TRV2:AcPDS*_*751*_, and *TRV2:AcPDS*_*1221*_) as measured by relative *AcPDS* transcript levels via qRT-PCR (*n* = 3); (B) Seedling survival rates recorded at 15 days post-inoculation (*n* = 6). **Supplementary Fig. S3**: Comparison of silencing efficiencies and seedling survival rates at 15 days post-inoculation of folded cotyledons of *A. canescens* via vacuum infiltration. (A) Silencing efficiency of TRV2: *AcPDS*-infected plants (TRV2: *AcPDS*_*311*_, *TRV2: AcPDS*_*751*_, and *TRV2: AcPDS*_*1221*_) as measured by relative *AcPDS* transcript levels via qRT-PCR (*n* = 3); (B) Seedling survival rates recorded at 15 days post-inoculation (*n* = 6). **Supplementary Table S1**: Primer sequences utilized for cloning the *AcPDS*,* AcTIP2;1 and AcPIP2;5* gene from *A. canescens*. **Supplementary Table S2**: Primer sequences employed in qRT-PCR analysis of *AcPDS*,* AcTIP2;1 and AcPIP2;5* in *A. canescens*

## Data Availability

No datasets were generated or analysed during the current study.
