# Supplementary material for: Establishment and optimization of a tobacco rattle virus -based virus-induced gene Silencing in Atriplex canescens
Source: Plant Methods. 2025 Aug 7;21:107. doi: 10.1186/s13007-025-01427-z (PMC12330136; doi:10.1186/s13007-025-01427-z)
Supplement: Supplementary file 1 — Supplementary Material 1: Supplementary Fig. S1: Isolation and characterization of the AcPDS gene from A. canescens. (A) Amplification of the full-length AcPDS cDNA sequence by nested PCR. (B) Amplification of three conserved AcPDS target fragments. (C) Multiple alignment of AcPDS protein with its homologs from other species. (D) Phylogenetic analysis of AcPDS and related homologs. Supplementary Fig. S2: Comparison of silencing efficiencies and seedling survival rates at 15 days post-inoculation of germinated A. canescens seeds via vacuum infiltration. (A) Silencing efficiency of TRV2:AcPDS-infected plants (TRV2:AcPDS311, TRV2:AcPDS751, and TRV2:AcPDS1221) as measured by relative AcPDS transcript levels via qRT-PCR (n = 3); (B) Seedling survival rates recorded at 15 days post-inoculation (n = 6). Supplementary Fig. S3: Comparison of silencing efficiencies and seedling survival rates at 15 days post-inoculation of folded cotyledons of A. canescens via vacuum infiltration. (A) Silencing efficiency of TRV2: AcPDS-infected plants (TRV2: AcPDS311, TRV2: AcPDS751, and TRV2: AcPDS1221) as measured by relative AcPDS transcript levels via qRT-PCR (n = 3); (B) Seedling survival rates recorded at 15 days post-inoculation (n = 6). Supplementary Table S1: Primer sequences utilized for cloning the AcPDS, AcTIP2;1 and AcPIP2;5 gene from A. canescens. Supplementary Table S2: Primer sequences employed in qRT-PCR analysis of AcPDS, AcTIP2;1 and AcPIP2;5 in A. canescens [file 13007_2025_1427_MOESM1_ESM.docx]

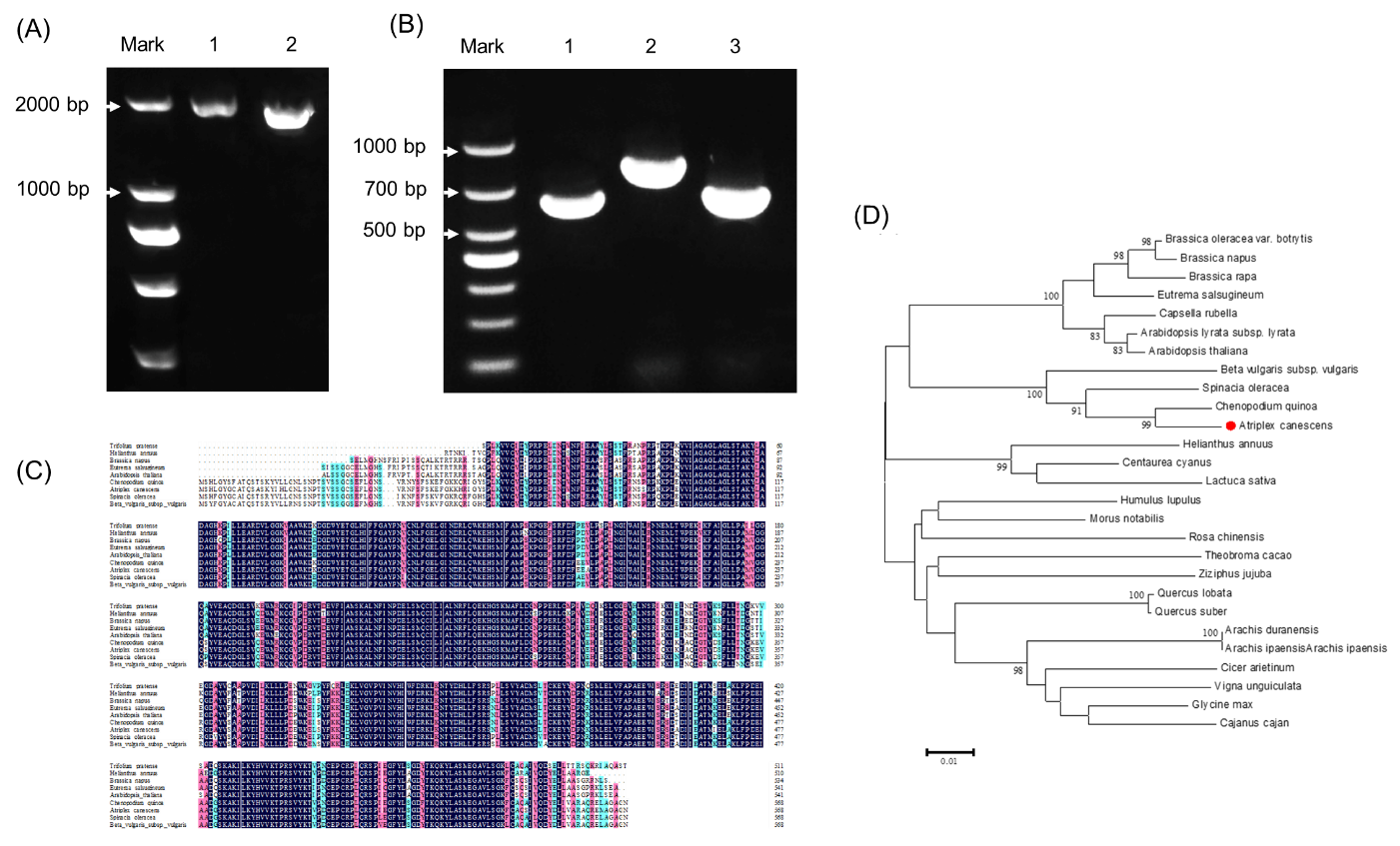


**Supplementary Fig. S1** Isolation and characterization of the *AcPDS* gene from *A. canescens*. (A) Amplification of the full-length *AcPDS* cDNA sequence by nested PCR. (B) Amplification of three conserved *AcPDS* target fragments. (C) Multiple alignment of AcPDS protein with its homologs from other species. (D) Phylogenetic analysis of AcPDS and related homologs.


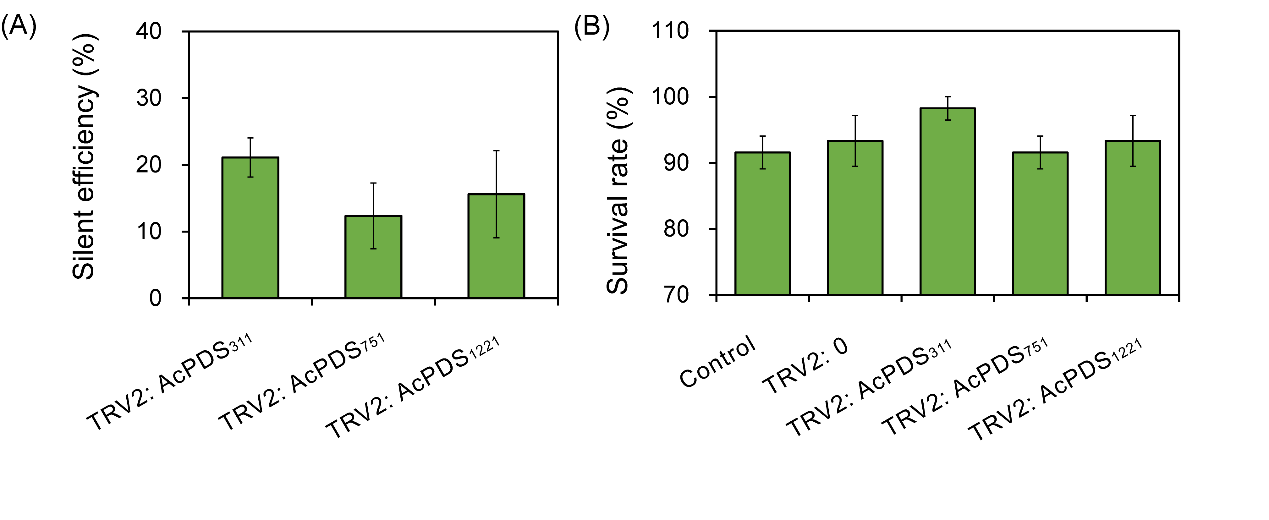


**Supplementary Fig. S2** Comparison of silencing efficiencies and seedling survival rates at 15 days post-inoculation of germinated *A. canescens* seeds via vacuum infiltration. (A) Silencing efficiency of TRV2:*AcPDS*-infected plants (TRV2:*AcPDS_311_*, *TRV2:AcPDS_751_*, and *TRV2:AcPDS_1221_*) as measured by relative *AcPDS* transcript levels via qRT-PCR (n = 3); (B) Seedling survival rates recorded at 15 days post-inoculation (n = 6).


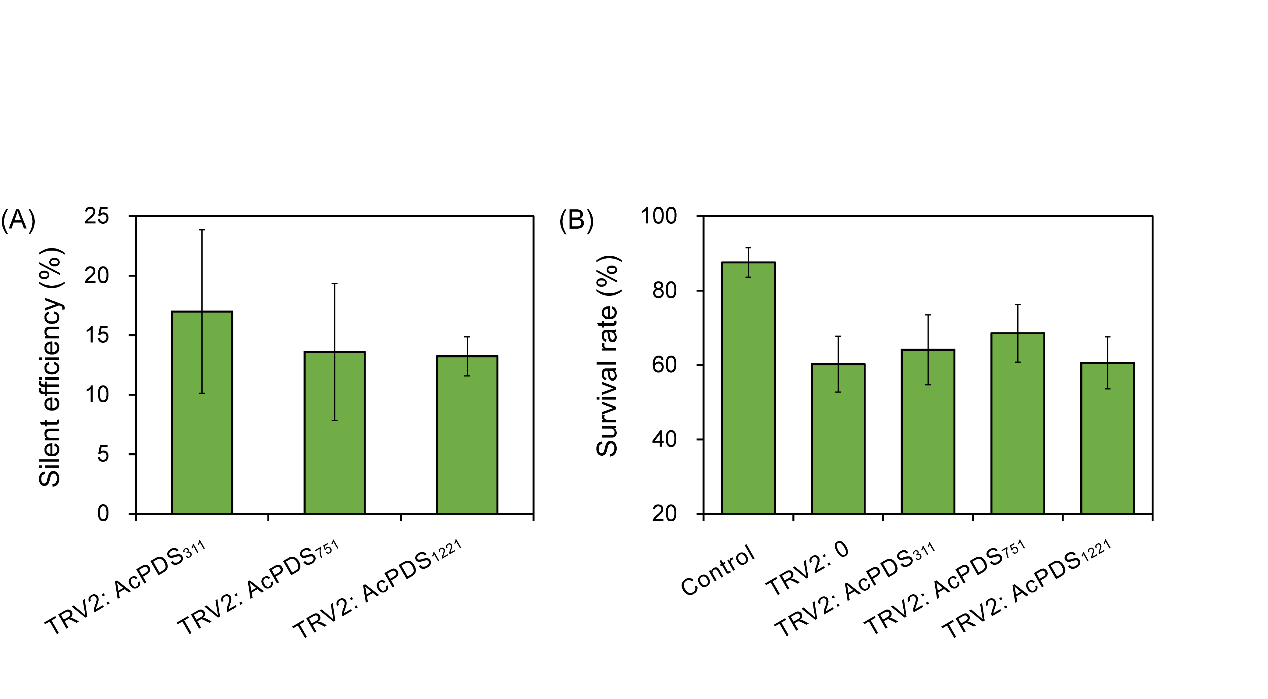


**Supplementary Fig. S3** Comparison of silencing efficiencies and seedling survival rates at 15 days post-inoculation of folded cotyledons of *A. canescens* via vacuum infiltration. (A) Silencing efficiency of TRV2:*AcPDS*-infected plants (TRV2:*AcPDS_311_*, *TRV2:AcPDS_751_*, and *TRV2:AcPDS_1221_*) as measured by relative *AcPDS* transcript levels via qRT-PCR (n = 3); (B) Seedling survival rates recorded at 15 days post-inoculation (n = 6).

**Supplementary Table S1** Primer sequences utilized for cloning the *AcPDS, AcTIP2;1 and AcPIP2;5* gene from *A. canescens*

| Primers | Sequences (5’-3’) |
| --- | --- |
| *AcPDS-*Outer-F | TCTCTCTCCTAGCTCACCTTG |
| *AcPDS-*Outer-R | CACCAATCCATACCTCCAATG |
| *AcPDS-*Inner-F | ATGAGTCATTTAGGATATGGTTGT |
| *AcPDS-*Inner-R | GTATTATACATTGCCTGCTCC |
| *AcPDS*_311_ | TAAGGTTACCGAATTGTGCTGGTTT  GGCTGGTCTAT |
| *AcPDS*_626_ | GCTCGGTACCGGATCCATATGCCATT  CAACGGTGC |
| *AcPDS*_751_ | TAAGGTTACCGAATTGTGGATGAG  GAAGCAAGGTGT |
| *AcPDS*_1104_ | GCTCGGTACCGGATCAACTGGAGCAG  CAGAGACATAAG |
| *AcPDS*_1221_ | TAAGGTTACCGAATTGAAAAACAC  ATACGACCATCTACT |
| *AcPDS*_1571_ | GCTCGGTACCGGATCTCCCCTGATAAA  TAGAAACCCTCT |
| *AcTIP2;1-*Outer-F | CTTCTCTCTTTCTTTCTTCAGC |
| *AcTIP2;1-*Outer-R | AAATTATACAACTTCAAACCCCTTT |
| *AcTIP2;1-*Inner-F | AAGCAATGGCAATTGCTTTTG |
| *AcTIP2;1-*Inner-R | TTTAGTATTCATTAGTGAGAGGG |
| *AcPIP2;5-*Outer-F | GGATCAATTCGAAGTTCATCAGTAC |
| *AcPIP2;5-*Outer-R | AATGCCTAGACACAAACGACTTAC |
| *AcPIP2;5-*Inner-F | ATGGCGAAAGATATAGAAGTAGG |
| *AcPIP2;5-*Inner-R | ATCACATGTTGGAGGAGCTTC |
| *AcTIP2;1*-F | TAAGGTTACCGAATTCATGCATTTGCCCTATTTGTTG |
| *AcTIP2;1*-R | GCTCGGTACCGGATCCCCAACAATGAATCCAATAGCAATA |
| *AcPIP2;5-*F | TAAGGTTACCGAATTCATGCTGCTGATCAATGCG |
| *AcPIP2;5-*R | GCTCGGTACCGGATCCCACTGCGAATCCAATAGGAAG |

**Supplementary Table S2** Primer sequences employed in qRT-PCR analysis of *AcPDS, AcTIP2;1 and AcPIP2;5* in *A. canescens*

| Primers | Sequences (5’-3’) |
| --- | --- |
| *AcPDS-*RT-F | TCAAGACTCCGAGGTCCGTT |
| *AcPDS-*RT-R | ACTTTTGCTTCGTGTAATCCCC |
| *AcTIP2;1*-RT-F | CAGGAGTAGGAGCAATAGAA |
| *AcTIP2;1*-RT-R | TGGCACCAACAATGAATC |
| *AcPIP2;5*-RT-F | CTCTATCTGACGGCTACA |
| *AcPIP2;5*-RT-R | GATGGTGGCTAAGTGAAC |
| *AcActin-F* | AAGAACTACGAGCTACCTGACGG |
| *AcActin-R* | GATACCAGAAGATTCCATTCCAAC |
